# Effects of creatine monohydrate supplementation and exercise on depression-like behaviors and raphe 5-HT neurons in mice

**DOI:** 10.20463/jenb.2016.09.20.3.4

**Published:** 2016-09-30

**Authors:** Nari Ahn, Yea Hyun Leem, Morimasa Kato, Hyukki Chang

**Affiliations:** 1 Laboratory of Exercise Physiology, Department of Human Movement Science, Seoul Women’s University, Seoul Republic of Korea; 2 Department of Neuroscience and TIDRC, Ewha Womans University Medical Center, Seoul Republic of Korea; 3 Department of Health and Nutrition, Yonezawa Nutrition University of Yamagata Prefecture, Yonezawa Japan

**Keywords:** Depression, Creatine, Exercise, Antidepressant, Behavior, 5-HT

## Abstract

**[Purpose]:**

The effects of creatine and exercise on chronic stress-induced depression are unclear. In the present study, we identified the effects of 4-week supplementation of creatine monohydrate and/or exercise on antidepressant behavior and raphe 5-HT expression in a chronic mild stress-induced depressed mouse model.

**[Methods]:**

Seven-week-old male C57BL/6 mice (n=48) were divided randomly into 5 groups: (1) non-stress control (CON, n=10), (2) stress control (ST-CON, n=10), (3) stress and creatine intake (ST-Cr, n=10), (4) stress and exercise (ST-Ex, n=9), and (5) combined stress, exercise, and creatine intake (ST-Cr+Ex, n=9). After five weeks’ treatment, we investigated using both anti-behavior tests (the Tail Suspension Test (TST) and the Forced Swimming Test (FST)), and 5-HT expression in the raphe nuclei (the dorsal raphe (DR) and median raphe (MnR)).

**[Results]:**

Stress for 4 weeks significantly increased depressive behaviors in the mice. Treatment with creatine supplementation combined with exercise significantly decreased depressive behaviors as compared with the CON-ST group in both the TST and FST tests. With stress, 5-HT expression in the raphe nuclei decreased significantly. With combined creatine and exercise, 5-HT positive cells increased significantly and had a synergic effect on both DR and MnR.

**[Conclusion]:**

The present study found that even a single treatment of creatine or exercise has partial effects as an antidepressant in mice with chronic mild stress-induced depression. Furthermore, combined creatine and exercise has synergic effects and is a more effective prescription than a single treatment.

## INTRODUCTION 

Due to various environmental stresses, people are increasingly suffering from various mental disorders such as depression, emotional instability, frustration and psychological internal injuries.^1^ In particular, patients with depressive disorder suffer from symptoms such as helplessness, increased anxiety, and decreased interest, appetite disorders, sleep disorders, and heart disease as well as suicidal impulses.^2^ Therefore, there is an urgent need for a multifaceted effort and research to solve these problems. 

The principal endocrine component of the stress response involves activation of the Hypothalamic-Pituitary-Adrenocortical (HPA) axis, which involves a neuroendocrine cascade culminating in the synthesis and secretion of glucocorticoids.^3^ In depression disorders caused by chronic stress, the relevant regulation of the corticotropin-releasing factors in the hypothalamus paraventricular nucleus^4^ is impaired and there is a negative feedback control of the HPA axis by corticosterons.^1^^,^^2^^,^^5^ The physiological causes of depression may be the lowering of brain serotonin (5-HT; 5-hydroxytryptamine) secretion.^6^ The 5-HT molecule is a monoamine neurotransmitter, like dopamine, adrenaline and noradrenaline and primarily found in the gastrointestinal tract, blood platelets, and the central nervous system. The neurons of the raphe nuclei (RN) are the principal source of 5-HT release in the brain. The dorsal raphe nuclei (DR) contains the largest number of serotonergic neurons; however, only 40% to 50% of the cell bodies in the DR are serotonergic.^7^ The 5-HT molecule is involved in a number of neuroendocrine features that appear in depression, such as emotions, arousal, aggressive behavior, surface activity, memory and learning, and stress response.^8^

Antidepressant medications including selective serotonin reuptake inhibitors (SSRIs), atypical antidepressants, tricyclic antidepressants, and monoamine oxidase inhibitors are generally regarded as effective treatments.^9^^,^^10^ However, antidepressants can sometimes cause a wide range of unpleasant side effects including nausea, increased appetite and weight gain, fatigue and drowsiness, sleep disturbance, suicide, and anxiety.^11^ Among modulators of stress management and depression without side effects, exercise has been effectively a plausible nonpharmacologic augmentation treatment.^12^^,^^13^ Exercise plays an effective antidepressant role in depression and also enhances secretion of the brain’s dopamine and beta-endorphins as well as 5-HT associated with mood regulation.^14^^-^^17^ Exercise is also a viable augmentation strategy for depressed patients who are nonresponsive to SSRIs.^13^

Four weeks of antidepressant tianeptine intake, with voluntary wheel running exercise, proved to be more effective in depression-related behavior experimental studies in the forced swim test, elevated plus maze, and open field test and the exercise group compared to the control group taking antidepressants demonstrated more positive behavior changes in the symptoms of depression.^5^^,^^18^^,^^19^ According to the movement and the study of the 5-HT expression in the brain for exercise intensity, a low-intensity physical movement is more positive in memory and learning than moderate-intensity and high-intensity exercise, and showed a positive side effect in movements.^20^ In a study conducted that combined aerobic exercise with antidepressant drugs (Sertraline), depression indicators improved more than in the antidepressants alone group or the control group,^21^ i.e., physical exercise is synergistic in maximizing the effects of medication. 

Creatine from food sources such as meat and fish has received attention as an effective nondrug antidepressant treatment. One of the most important physiological functions of creatine relates to the energy levels in muscle and brain tissue.^22^ Creatine, synthesized adenosine triphosphate (ATP), is a major source of energy, and has been primarily known as a supplement for muscle power and to improve sports performance.^23^ Creatine intake produces phosphocreatine (PC), which is attached to a phosphate molecule, and partially forms the ATP-PC energy system and free creatine, and increases the body’s stores of creatine in the muscle to provide more immediate energy.^24^^-^^26^ Hence, creatine supplementation increases muscle strength and physical performance in traditional sports.^27^

Moreover, in a recent study, creatine intake had an effective role in learning, memory, power state, emotional, and cognitive function.^28^ Creatine intake improved the antidepressive-behavior in animal models^29^^-^^31^ and working memory scores^28^ as well as the Hamilton Depression Rating Scale (HAM-D) score in humans.^32^ In another recent study, treatment combined with creatine and antidepressants was effective in depressed patients. Receiving both creatine supplementation and escitalopram has been shown to improve depression symptoms.^33^ However, the effect of psychologic and physiologic factors as antidepressants on antidepressant behavior and brain 5-HT levels of creatine intake and/or exercise is unclear. In particular, creatine supplementation has not been known for synergies as compared to each treatment only in combination with exercise. To resolve these issues in the present study, we determined whether treatment combining an exercise regimen and/or creatine supplementation improves antidepressant behavior and raphe 5-HT levels in chronic mild stress-induced depressed mice. 

## METHODS 

### Experimental animals 

Seven-week-old male C57BL/6 mice (n=48) were housed in cages illuminated from 07:00 to 19:00 (12:12 h cycle) with room temperature varying from 22 to 24℃. We supplied sufficient feed and water. All procedures were performed in accordance with the Institutional Guidelines for Animal Care at the National Institutes of Health Guidelines for the Care and Use of Laboratory Animals and the deliberations of the Research Ethics Committee of the Seoul Women’s University. 

Experimental groups were divided into non-stress control group (CON, n=10), stress control group (ST-CON, n=10), stress and creatine intake group (ST-Cr, n=10), stress and exercise group (ST-Ex, n=9), and Combined stress, exercise and creatine intake group (ST-Cr+Ex, n=9). 

### Creatine supplement 

The creatine dose was created by mixing a pellet of creatine monohydrate (Sigma chemical; 0 kcal/g) corresponding to a 4% intake-volume of the normal diet to provide a pellet.^29^^,^^31^ To identify the intake volume of creatine and chow, we identified the relevant amounts daily in all groups. 

### Experimental design and exercise protocol 

To identify the antidepressant effects of creatine intake, we applied chronic mild stress (CMS), and after 2 days, we performed a forced swim test for 15 min in all mice except the control group.^34^ The CMS consisted of 3 different and sequential stress situations as follows: 1) inclining their cage by 20° from the horizontal for 48 hr; 2) wetting their chip bedding with 200 ml of water for 24 hr; 3) agitating the cages at 180 rpm by a rotatory shaker for 24 hr (normal cage for 24 hr between each situation). We repeated these stress situations for the mice for a period of 4 weeks. The administration of creatine and exercise were the treatments provided for 4 weeks commencing 1 week after CMS was applied. Exercise groups (ST-Ex and ST-Cr+Ex) utilized a treadmill running for 50 min (5 m/min for 10 min, 8 m/min for 30 min, 5 m/min for 10 min, 0% grade). Exercise was conducted as often as 5 days per week at 18:00-20:00. Treadmill speed was increased gradually so as not to increase the stress to the experimental animals. 

### Behavior analysis 

#### Forced Swimming Test 

To assess depression related behavior, the mice were placed into a Plexiglas cylinder (height 25 cm × 15 cm diameter) filled up to 20 cm with water at 24–26 ℃, and forced swim for 4 min (modified by Porsolt et al.(10)). We measured immobilizing time with the video camera for 4 min. 

#### Tail Suspension Test 

In brief, each mouse was suspended on the edge of a rod 50 cm above the floor in a visually isolated area by adhesive tape placed approximately 1 cm from the tip of the tail. We measured immobilizing time with the video camera for 4 min. 

#### Tissue preparation and immunohistochemistry 

For immunohistochemical (IHC) analysis, the mice were anesthetized with 2,2,2-tribroeothanol (i.p.) and perfused them transcardially with 100 ml of 0.9% saline. After perfusion, the brains were quickly dissected out and fixed for 48 h at 4℃ in 4% paraformaldehyde in 0.1 M phosphate buffer (pH 7.4) and immersed in 30% sucrose solution. Fixed brain tissues were stored at -80℃. Frozen serial frontal sections (30-μm-thick) of the brain were made using a cryomicrotome (Leica, Germany). 

A sensitive IHC method employing a free floating technique was used, as previously performed by our group.^35^ We used a specific antibody (Immunostar Inc.): anti-5-HT rabbit (1:20,000) to identify 5-HT activity and used diaminobenzidine dihydrochloride (DAB) as the chromogen for visualization. After the sections were mounted and viewed under a microscope, the stained cells were assessed with photo images (at 100× resolution) using the Image J program (NIH Image Engineering, Bethesda, MD). 

#### Statistical analysis 

All the data were represented as mean ± standard error and IBM Statistics 20 was used for all the statistical analysis in this study. One-way ANOVA was conducted to understand differences based on each treatment, and a post hoc test was conducted with Fisher’s LSD for the variables that showed a significant difference. The significance was accepted at the level of p<0.05. 

**Figure 1.**
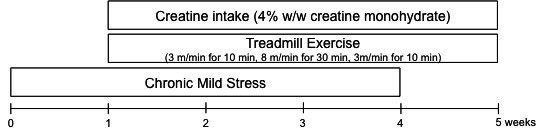
The experimental design. This study was conducted over a period of treatment of 5 weeks. From week 2 of CMS treatment, exercise and creatine treatments were performed over 4 weeks.

## RESULTS 

### Tail suspension test 

**Figure 2.**
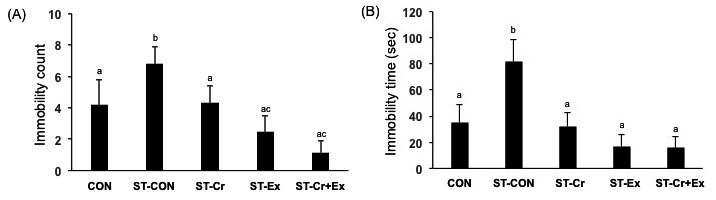
The data of tail suspension test during 4 min. (A) Immobility count (B) Immobility time. Letters a, b, and c indicate significant differences.

Figure 2 shows the antidepressant behavior during the tail suspension test including immobility count and time. The immobility counts revealed that the ST-CON (4.20 ± 1.57) group increased, but not significantly compared with the CON (6.80 ± 1.12) group (Fig. 2A). The ST-Ex (2.44 ± 1.04, p<0.05) and ST-Cr+Ex (1.11 ± 0.77, p<0.01) groups had significantly reduced immobility counts compared with the ST-CON group. Immobility time (seconds) in the ST-CON (81.90 ± 16.57, p<0.01) group was significantly increased compared with the CON (35.30 ± 13.84) group (Fig 2B). In the ST-Cr (32.20 ± 10.12, p<0.01), ST-Ex (16.78 ± 8.91, p<0.01), and ST-Cr+Ex (15.67 ± 8.44, p<0.01) groups immobility time was significantly decreased compared to the ST-CON group. 

### Forced swimming test 

**Figure 3.**
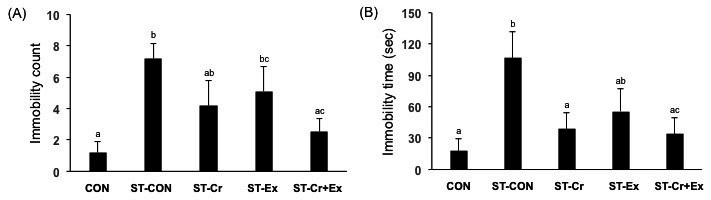
The data of forced swimming test over 4 min. (A) Immobility count (B) Immobility time. Letters a, b, and c indicate significant differences.

Figure 3 shows antidepressant behavior during the forced swimming test including immobility count and time. The immobility counts revealed that in the ST-CON (7.20 ± 0.95) group the immobility count significantly increased compared to the CON (1.20 ± 0.73, p<0.01) group (Fig. 3A), and the ST-Cr+Ex (2.56 ± 0.84, p<0.01) group had a significantly reduced immobility count compared to the ST-CON group. However, the ST-Cr (4.20 ± 1.58) and ST-Ex (5.11 ± 1.55) groups were not significantly affected compared with the ST-CON group. Immobility time (seconds) in the ST-CON (106.70 ± 24.86, p<0.01) group was significantly increased compared to the CON (17.90 ± 11.70) group (Fig. 3B). The ST-Cr (39.00 ± 14.98, p<0.05) and ST-Cr+Ex (34.11 ± 15.48, p<0.01) groups had significantly decreased counts compared to the ST-CON group, but, the ST-Ex (72.22 ± 25.99) group was not significantly affected as compared to the ST-CON group. 

### 5-HT expression in dorsal raphe nucleus 

**Figure 4.**
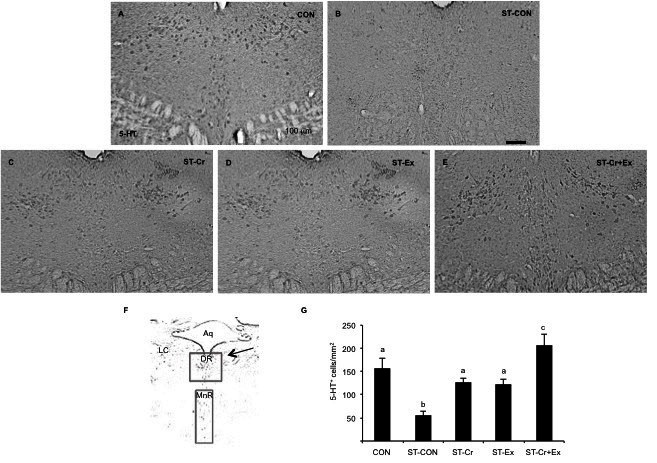
5-HT expression in the dorsal raphe nucleus. (A) Control group (B) Stress group (C) combined stress and creatine intake group (D) combined stress and exercise group (E) combined stress, creatine intake and exercise group (F) the brain map. Aq, cerebral aqueduct; DR, dorsal raphe nucleus; LC, locus coeruleus; MnR, median raphe nucleus (G) bar graphs summarizing the 5-HT expression.

As shown in Fig. 4, 5-HT expression in the DR was significantly decreased in the ST-CON (55.00 ± 8.66, p<0.01) group compared with the CON (157.50 ± 22.2) group. The ST-Cr (126.79 ± 8.93, p<0.05), ST-Ex (123.57 ± 10.70, p<0.05), and ST-Cr+Ex (206.07 ± 24.38, p<0.01) groups showed significantly increased 5-HT cell expression compared to the ST-CON group. Indeed, the ST-Cr+Ex group had a synergic effect that significantly increased 5-HT cell expression compared to the ST-Cr (p<0.05) and ST-Ex groups (p<0.01). 

### 5-HT expression in median raphe nucleus 

**Figure 5.**
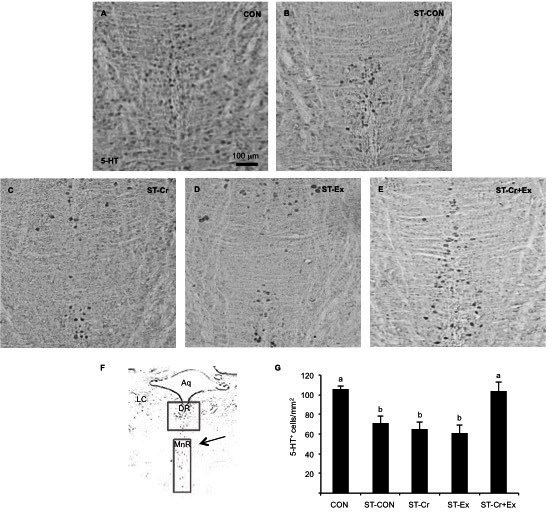
5-HT expression in the median raphe nucleus. (A) Control group (B) Stress group (C) combined stress and creatine intake group (D) combined stress and exercise group (E) combined stress, creatine intake and exercise group (F) the brain map. Aq, cerebral aqueduct; DR, dorsal raphe nucleus; LC, locus coeruleus; MnR, median raphe nucleus (G) bar graphs summarizing the 5-HT expression.

As shown in Fig. 5, the 5-HT expression in the MnR significantly decreased in the ST-CON (71.07 ± 7.38, p<0.05) group compared to the CON (105.35 ± 3.34) group. The ST-Cr (65.00 ± 7.50) and ST-Ex (60.71 ± 8.05) groups did not show any change in 5-HT cell expression compared to the ST-CON group. The ST-Cr+Ex (103.93 ± 8.88) group only increased 5-HT expression in the MnR compared to the ST-CON group (p<0.05). The ST-Cr+Ex group showed a synergic effect compared to the ST-Cr (p<0.01) and ST-Ex groups (p<0.01). 

## DISCUSSION 

Modern society is surrounded by many environmental stresses, and prolonged stress may result in depressive disorders. The present study evaluated 5-HT expression of the RN and utilized a behavior test to evaluate the antidepressant effects due to creatine intake and/or exercise in mice. This study is the first to examine the effects of creatine and/or exercise on behavior and 5-HT cell expression using the CMS depression model. According to the results of previous experiments which used the CMS method, CMS resulted in increased CRH mRNA and a decrease of glucocorticoid receptor (GR) protein expression in the PVN.^36^ Therefore, the CMS model used in this study established the depressive mouse model related to activation of the HPA axis. To provide a stress environment at a particular time, creatine intake and exercise groups were treated simultaneously with CMS after 1 week of pre-treatment (CMS begins to appear in the depression phase, according to our data). Creatine supplementation was mixed with chow to an intake of approximately 4% w/w creatine monohydrate as in previous work,^29^^,^^31^ the mixing rate having been calculated in our preliminary study. Mild exercise was applied without the influence of negative stress, as forced exercises like the treadmill are stressful in mice, depending on speed or intensity. 

There was no difference in the intake volume and weight between pre-test and post-test of the groups, consistent with the results that 4% w/w creatine intake did not affect stress.^31^^,^^37^ In this study, we used TST and FST to analyze antidepressant behavior. In both tests, creatine intake showed antidepressant behavior. The ST-CON group had significantly higher immobility time than the CON group in TST (immobility count was not significant). Both ST-Cr and ST-Ex groups had significantly lower immobility times than the ST-CON group (the ST-Ex group appears lower). However, there was no synergic effect in the ST-Cr+Ex group, consistent with the antidepressant behavior result of TST on subcutaneous administration of creatine.^30^ The ST group had a significantly higher immobility count and time in the FST than the CON group; the immobility count and time were, however, significantly decreased in the ST-Cr and ST-Cr+Ex group except for the ST-Ex group. These probably did not affect the antidepressant behaviors because of the mild intensity of the exercise. In an animal study, creatine intake showed improved antidepressant activity in neurobehavioral tests, which suggests a neuroprotective role and promotion of cell growth.^38^^,^^39^ In human studies, creatine intake over 4 weeks (3-5g/day) showed antidepressant effects in tests for depression and anxiety.^32^ In addition creatine plus the SSRI escitalopram improved the depressive symptoms and provided superior efficacy, relatively good tolerability, and minimal side effects in depressive disorders.^33^ Specifically, exercise increases 5-HT synthesis and metabolism as well as beta-endorphins to improve the clinical efficacy of exercise treatment of depression and anxiety disorders.^40^^,^^41^ However, in our data the antidepressant behavior of ST-Ex group was weaker (not significant) than the ST-Cr group in FST, likely due to the mild exercise intensity to obtain an optimal antidepressant effect. According to studies on the relation between exercise intensity (mild, moderate, high) and depression, mild and moderate intensity was sufficiently effective to reduce depression.^42^^,^^43^ Spontaneous wheel running exercise improves the depression behavioral consequences of stressor exposure, which may be even more protective than forced exercise that fails to alter behavior in some models.^44^ Thus, our investigation found mild treadmill exercise to be superior to high intensity exercise that can be stressful over a long period. 

The neurotransmitter 5-HT plays an important role in depression, sensory perception, sleepiness, and mood. In the present study, we measured the 5-HT positive cell in the RN (DR and MnR) with antidepressant behavioral tests. CMS over 4 weeks was sufficient to reduce the 5-HT in the RN, resulting in persistent deficits in presysnaptic mechanisms that control 5-HT.^45^ Our treadmill exercise enhanced the levels of 5-HT in DR, similar to the result of low speed exercise, but not high speed, significantly increasing c-Fos expression in 5-HT neurons in the DR compared to the control.^46^ The exercise effect results from increases of tryptophan hydroxylase, which is the rate-limiting enzyme of 5-HT biosynthesis.^47^ However, the effect of creatine supplementation on the 5-HT is unclear. 

Both ST-Cr and ST-Ex groups had significantly increased 5-HT positive cells compared to the ST-CON group in the DR. Further, the ST-Cr+Ex group showed a synergistic effect greater than in the ST-Cr and ST-Ex groups. However, the changes in the MnR showed a different pattern, where the 5-HT-positive cells of ST-Cr and ST-Ex groups did not increase in comparison to the ST-CON group, and only showed significant increase in the ST-Cr+Ex group. The MnR is known as the part associated with hallucinations.^48^ Persons who have major clinical depression have been reported to experience hallucinations and delusions.^49^ These results suggest that the combination of creatine and exercise would produce an improved antidepressant effect. 

Despite the brain comprising only about 2% of the body, the brain uses about 20% of total energy of the body. This suggests that the brain’s energy supply is extremely important, and the physiological energy of our body is related to ATP turnover. The ATP synthesis rate is counterbalanced against ATP consumption for optimal performance. Creatine metabolism and the creatine kinase/PC system are important for normal brain function, and may be compromised in diseases of the central nervous system.^50^ Creatine administration increases creatine concentrations and thus the production of neurotransmitters and PC in the brain,^39^ which have both neurological and hormonal effects on the body and make the brain less susceptible to experiencing depression. In addition, creatine administration, similar to the effect of exercise, has also been shown to have neuroprotective effects in brain health including the delay of the progression of neurodegeneration in Huntington’s, Alzheimer’s and Parkinson’s disease.^51^ Although treatment with either creatine or exercise is effective as an antidepressant, the combination of creatine and exercise has a synergic effect, which is a more effective prescription than either treatment by itself. 
